# Supplementary material for: Anti-inflammatory, analgesic, antioxidant, and alpha-amylase inhibitory effects of the hydroethanolic leaf extract of Aleuritopteris bicolor (Roxb.) Fraser-Jenk
Source: PLoS One. 2025 Jun 23;20(6):e0326808. doi: 10.1371/journal.pone.0326808 (PMC12184904; doi:10.1371/journal.pone.0326808)
Supplement: Attachment — Submitted filename: PONE-D-24-47035_reviewer_ 3rd paper.pdf [file pone.0326808.s001.pdf]

PLOS ONE

Ethanolic Extract of Aleuritopteris bicolor Leaves: A Promising Natural Agent for Pain Management, Inflammation Control, and Diabetes Mitigation in Albino Rats

--Manuscript Draft--

**Manuscript Number:**	PONE-D-24-47035
**Article Type:**	Research Article
**Full Title:**	Ethanolic Extract of Aleuritopteris bicolor Leaves: A Promising Natural Agent for Pain Management, Inflammation Control, and Diabetes Mitigation in Albino Rats
**Short Title:**	Biological activity of Aleuritopreris bicolor leaves
**Corresponding Author:**	Bipindra Pandey, Master<br>Madan Bhandari Academy of Health Sciences<br>Hetauda, Bagamati NEPAL
**Keywords:**	Aleuritopteris bicolor; Analgesic; Anti-inflammatory; Alpha-amylase
**Abstract:**	Aleuritopteris bicolor (A. bicolor) leaves have been traditionally used for treating stomach discomfort and aiding wound healing.This study aimed to investigate the anti-inflammatory, analgesic, and alpha-amylase inhibitory properties of A. bicolor leaves from Nepal.The flavonoid and phenolic contents of A. bicolor leaf ethanolic extract were quantified using the aluminum chloride and Folin-Ciocalteu methods. Antioxidant activity was assessed via FRAP, H2O2, DPPH, and NO scavenging assays, whereas anti-inflammatory and analgesic effects were evaluated using carrageenan-induced paw edema in rats and tail flick/hot plate tests, respectively. The alpha-amylase inhibitory activity was also investigated.The extract exhibited high flavonoid (405.95 ± 0.28 mg QE/g) and phenolic content (20.98 ± 0.20 mg GAE/g), strong antioxidant activity (IC50:9.87 µg/mL, DPPH; 249.59 µg/mL, H2O2; 72.98 µg/mL, NO), and significant reducing power (0.51 ± 0.004). It increased analgesic activity by 38.89% (hot plate test) and 16.84% (tail-flick test) at 250 mg/kg, reduced inflammation by 36.44% at 180 min (500 mg/kg), and inhibited alpha-amylase activity (IC50:59.31 µg/mL). A. bicolor exhibits significant analgesic, anti-inflammatory, and hypoglycemic potential, making it a promising candidate for further pharmacological exploration.
**Order of Authors:**	Prabhat Kumar Jha, M. Pharm<br>Bipindra Pandey, M. Pharm<br>Ram Kishor Yadav, Bachelor<br>Sindhu KC, M. Pharm<br>Sandesh Poudel, Bachelor<br>Sushil Panta, PhD
**Additional Information:**	
**Question**	**Response**
**Financial Disclosure**<br><br>Enter a financial disclosure statement that describes the sources of funding for the work included in this submission. Review the submission guidelines for detailed requirements. View published research articles from *PLOS ONE* for specific examples.<br><br>This statement is required for submission	The author(s) received no specific funding for this work.

Powered by Editorial Manager® and ProduXion Manager® from Aries Systems Corporation

and **will appear in the published article** if the submission is accepted. Please make sure it is accurate.<br><br>**Funded studies**<br>Enter a statement with the following details:<br>• Initials of the authors who received each award<br>• Grant numbers awarded to each author<br>• The full name of each funder<br>• URL of each funder website<br>• Did the sponsors or funders play any role in the study design, data collection and analysis, decision to publish, or preparation of the manuscript?<br><br>Did you receive funding for this work?	
**Competing Interests**<br><br>Use the instructions below to enter a competing interest statement for this submission. On behalf of all authors, disclose any competing interests that could be perceived to bias this work—acknowledging all financial support and any other relevant financial or non-financial competing interests.<br><br>This statement is required for submission and **will appear in the published article** if the submission is accepted. Please make sure it is accurate and that any funding sources listed in your Funding Information later in the submission form are also declared in your Financial Disclosure statement.<br><br>View published research articles from *PLOS ONE* for specific examples.	The authors have declared that no competing interests exist.

**NO authors have competing interests**<br><br>Enter: *The authors have declared that no competing interests exist.*<br><br>**Authors with competing interests**<br><br>Enter competing interest details beginning with this statement:<br><br>*I have read the journal's policy and the authors of this manuscript have the following competing interests: [insert competing interests here]*<br><br>* typeset	
**Ethics Statement**<br><br>Enter an ethics statement for this submission. This statement is required if the study involved:<br><br>• Human participants<br>• Human specimens or tissue<br>• Vertebrate animals or cephalopods<br>• Vertebrate embryos or tissues<br>• Field research<br><br>Write "N/A" if the submission does not require an ethics statement.<br><br>General guidance is provided below. Consult the submission guidelines for detailed instructions. **Make sure that all information entered here is included in the Methods section of the manuscript.**	The research was conducted in compliance with the National Institutes of Health (NIH) guidelines for the Care and Use of Laboratory Animals. The Institutional Review Committee (IRC) at the Pokhara University Research Center (PURC) on the Ethics of Animal Experiments reviewed and approved the study protocol (Protocol Number: 105/079/80). During all research involving animal subjects was carried out in accordance with the regulations and guidelines established by NIH and every effort was made to reduce animal suffering.

**Format for specific study types**<br><br>**Human Subject Research (involving human participants and/or tissue)**<br>• Give the name of the institutional review board or ethics committee that approved the study<br>• Include the approval number and/or a statement indicating approval of this research<br>• Indicate the form of consent obtained (written/oral) or the reason that consent was not obtained (e.g. the data were analyzed anonymously)<br><br>**Animal Research (involving vertebrate animals, embryos or tissues)**<br>• Provide the name of the Institutional Animal Care and Use Committee (IACUC) or other relevant ethics board that reviewed the study protocol, and indicate whether they approved this research or granted a formal waiver of ethical approval<br>• Include an approval number if one was obtained<br>• If the study involved *non-human primates*, add *additional details* about animal welfare and steps taken to ameliorate suffering<br>• If anesthesia, euthanasia, or any kind of animal sacrifice is part of the study, include briefly which substances and/or methods were applied<br><br>**Field Research**<br><br>Include the following details if this study involves the collection of plant, animal, or other materials from a natural setting:<br>• Field permit number<br>• Name of the institution or relevant body that granted permission	
**Data Availability**<br><br>Authors are required to make all data underlying the findings described fully available, without restriction, and from the time of publication. PLOS allows rare exceptions to address legal and ethical concerns. See the PLOS Data Policy and FAQ for detailed information.	Yes - all data are fully available without restriction

A Data Availability Statement describing where the data can be found is required at submission. Your answers to this question constitute the Data Availability Statement and **will be published in the article**, if accepted.<br><br>**Important:** Stating 'data available on request from the author' is not sufficient. If your data are only available upon request, select 'No' for the first question and explain your exceptional situation in the text box.<br><br>Do the authors confirm that all data underlying the findings described in their manuscript are fully available without restriction?	
**Describe where the data may be found in full sentences. If you are copying our sample text, replace any instances of XXX with the appropriate details.**<br><br>• If the data are **held or will be held in a public repository**, include URLs, accession numbers or DOIs. If this information will only be available after acceptance, indicate this by ticking the box below. For example: *All XXX files are available from the XXX database (accession number(s) XXX, XXX.*).<br>• If the data are all contained **within the manuscript and/or Supporting Information files**, enter the following: *All relevant data are within the manuscript and its Supporting Information files.*<br>• If neither of these applies but you are able to provide **details of access elsewhere**, with or without limitations, please do so. For example:<br><br>*Data cannot be shared publicly because of [XXX]. Data are available from the XXX Institutional Data Access / Ethics Committee (contact via XXX) for researchers who meet the criteria for access to confidential data.*<br><br>*The data underlying the results presented in the study are available from (include the name of the third party*	All study data and materials will be made available to the corresponding author upon request.

Powered by Editorial Manager® and ProduXion Manager® from Aries Systems Corporation

and contact information or URL).<br>• This text is appropriate if the data are owned by a third party and authors do not have permission to share the data.<br><br>* typeset	
Additional data availability information:	

Manuscript

Click here to access/download;Manuscript;AB_Manuscript-PLOS.doc

Research Article

Ethanolic Extract of *Aleuritopteris bicolor* Leaves: A Promising Natural Agent for Pain Management, Inflammation Control, and Diabetes Mitigation in Albino Rats

Prabhat Kumar Jha[1, 2], Bipindra Pandey[*1, 2], Ram Kishor Yadav[2], Sindhu KC[3], Sandesh Poudel[2], Sushil Panta[2]

[1]Department of Pharmacy, Madan Bhandari Academy of Health Sciences, Hetauda, Nepal

[2]School of Health and Allied Sciences, Pokhara University, Pokhara, Nepal

[3]Chitwan Medical College, Tribhuvan University, Chitwan, Nepal

***Corresponding author:**

E-mail: bipindra.pandey@mbahs.edu.np (BP)

Short Title: Biological activity of *Aleuritopreris bicolor* leaves

Abstract

Aleuritopteris bicolor (*A. bicolor*) leaves have been traditionally used for treating stomach discomfort and aiding wound healing.This study aimed to investigate the anti-inflammatory, analgesic, and alpha-amylase inhibitory properties of *A. bicolor* leaves from Nepal.The flavonoid and phenolic contents of *A. bicolor* leaf ethanolic extract were quantified using the aluminum chloride and Folin-Ciocalteu methods. Antioxidant activity was assessed via FRAP, H_2O_2, DPPH, and NO scavenging assays, whereas anti-inflammatory and analgesic effects were evaluated using carrageenan-induced paw edema in rats and tail flick/hot plate tests, respectively. The alpha-amylase inhibitory activity was also investigated.The extract exhibited high flavonoid (405.95 ± 0.28 mg QE/g) and phenolic content (20.98 ± 0.20 mg GAE/g), strong antioxidant activity (IC_{50}:9.87 µg/mL, DPPH; 249.59 µg/mL, H_2O_2; 72.98 µg/mL, NO), and significant reducing power (0.51 ± 0.004). It increased analgesic activity by 38.89% (hot plate test) and 16.84% (tail-flick test) at 250 mg/kg, reduced inflammation by 36.44% at 180 min (500 mg/kg), and inhibited alpha-amylase activity (IC_{50}:59.31 µg/mL). *A. bicolor* exhibits significant analgesic, anti-inflammatory, and hypoglycemic potential, making it a promising candidate for further pharmacological exploration.

Keywords: *Aleuritopteris bicolor*, Analgesic, Anti-inflammatory, Alpha-amylase

Introduction

Inflammation is the process of tissue repair after injury or damage. This process consists of a sequence of cellular and microvascular reactions aimed at clearing damaged tissues and promoting the formation of new tissues [1]. This leads to increased vascular permeability and enhanced blood flow, resulting in congestion and thrombosis. Leukocyte migration is accompanied by tissue destruction via proteolytic activity, necrosis, and apoptosis. This is followed by the depletion of phagocytic cells, production of new humoral mediators that stimulate cell proliferation, and regeneration of functional and connective tissues. The final phase of inflammation is termed resolution of inflammation [2]. Inflammation can be broadly classified into acute and chronic inflammation, although the cause of inflammation varies, such as external and internal factors. External factors can be non-microbial (toxins, allergens, irritants) and microbial (virulence factors, pathogen-associated molecular patterns). Diseases associated with chronic inflammation include cardiovascular disease, arthritis, diabetes, bowel disease, liver disease, and cancer [3]. Pain is a feeling of discomfort that varies from one individual to another. Pain ranges from perceptible to mild to intense and overwhelming. Pain can be indicated by sensations such as pricking, tingling, and stinging, burning, shooting, aching, or electric feelings [4]. Non-steroidal anti-inflammatory drugs (NSAIDs) and opioids are commonly used to treat inflammatory diseases and manage pain (8). NSAIDs, the primary drugs used for inflammation, are effective inhibitors of cyclooxygenase (COX). Initially, these drugs were regarded as safe, but subsequent literature reports have revealed adverse reactions [5]. The most notable side effects of NSAIDs are gastrointestinal infections and kidney complications, which are primarily attributed to the free COOH group in their structure [6]. Diabetes, a widespread metabolic disorder, affects millions of people worldwide [7]. The management of diabetes requires a multifaceted approach, including lifestyle changes, medication, and insulin therapy. Lifestyle interventions, such as adopting a

healthy diet, engaging in regular physical activity, and maintaining a healthy weight, can help regulate blood sugar levels and reduce the risk of complications [8]. One strategy for managing diabetes is to reduce postprandial glycemia by inhibiting the activity of alpha-glucosidase and alpha-amylase, which are responsible for carbohydrate hydrolysis. Additionally, pharmacological treatments, including oral hypoglycemic agents and insulin, may be necessary to achieve optimal glycemic control. Given its complexity, diabetes requires an integrated approach for its effective control and management[9]. *Aleuritopteris bicolor*, a fern from the Pteridaceae family, is native to the Indian subcontinent. Local populations have traditionally used various parts of this plant to treat a range of ailments [10]. The stem has been documented for its wound healing and antipyretic properties, whereas the leaves are commonly used to address gastritis-related issues [11]. However, limited pharmacological studies have been conducted on *A. bicolor* leaves. This study presents preliminary findings on the analgesic and anti-inflammatory effects of *A. bicolor* leaves, as well as their *In vitro* α-amylase inhibitory activity, to explore the potential of *A. bicolor* as a multitarget therapeutic approach and its prospective pharmaceutical applications.

Materials and methods

Plant Material and Preparation of *A. bicolor* leaves extract

Leaves of *Aleuritopteris bicolor* were collected from Pokhara-31 in the Kaski district of Nepal and identified by comparing with relevant literature and through verification at the National Herbarium and Plant Laboratory, Godavari, Lalitpur. The plant specimen, stored in the Pharmacognosy Laboratory of Pokhara University under Herbarium Sheet registration number PUH-2022-39, was thoroughly washed, shade-dried, and then powdered. A modified cold maceration method was used for extraction: 100 g of the powdered material was macerated in 800 ml ethanol for 24 hours. This process was repeated, and the filtrates were

collected and concentrated using a rotary evaporator under controlled conditions (175-250 mbar pressure, 90 rpm, 40°C water bath, and 5°C chilling). The dried extracts were stored in a vacuum desiccator at 60 mbar pressure until fully stabilized [12].

Phytochemical analysis of *A. bicolor* leaves extract

Preliminary phytochemical screening of *A. bicolor* leaves extracts

The extracts from *Aleuritopteris bicolor* leaves were subjected to phytochemical analysis to detect the presence of secondary metabolites, including alkaloids, flavonoids, tannins, carbohydrates, anthraquinones, saponins, and proteins [13].

Estimation of Phenolic and Flavonoid content in *A. bicolor* leaves extracts

The total phenolic content (TPC) of *Aleuritopteris bicolor* extracts was measured using the Folin-Ciocalteu method, with the results expressed in milligrams of gallic acid equivalents per gram of dry leaf extract (mg GAE/g). The total flavonoid content (TFC) was determined using the aluminum chloride method, and the results were expressed in milligrams of quercetin equivalents per gram of dry leaf extract (mg QE/g) [14].

In vitro Antioxidant Activity of *A. bicolor*

In vitro DPPH Free Radical Scavenging Assay

The DPPH radical scavenging activity of the plant extracts was assessed by combining 2 ml of the extract or ascorbic acid (ranging from 1 to 60 µg/mL) with 2 ml of 100 µM DPPH in ethanol. After allowing the mixture to stand in the dark for 30 minutes, the absorbance was measured at 517 nm using a UV spectrophotometer, and the IC_{50} value was subsequently calculated [15].

$$\text{Radical Scavenging Activity (\%)} = \frac{A_0 - A_s}{A_0} * 100\%$$

Where Ao, represents the absorbance of the control and As denotes the absorbance of the

sample. The control refers to the test solution that does not contain a sample, while ascorbic acid served as the positive control. The antioxidant activity of each sample was quantified as the IC_{50}, which indicates the concentration needed to inhibit DPPH radical formation by 50%. This value was calculated from the graph by plotting the percentage inhibition against the concentration of the extract.

Nitric Oxide Scavenging Activity

Nitric oxide radical scavenging activity was evaluated by incubating sodium nitroprusside (SNP) with plant extracts (ranging from 0.1 to 1000 µg/mL) at pH 7.2 for 2.5 hours at 29°C. Nitrite formation was determined by adding Griess reagent, and the absorbance was measured at 548 nm using a UV spectrophotometer [16]. The radical scavenging activity of the extract was calculated using a specific formula:

$$\text{Nitric Oxide Scavenging Activity (\%)} = \frac{A_0 - A_s}{A_0} * 100\%$$

Where Ao represents the absorbance of the control and As denotes the absorbance of the sample. The control refers to the test solution that does not contain a sample, with curcumin serving as the positive control. A similar procedure was conducted using curcumin solutions at various concentrations: 0.1 µg/mL, 1 µg/mL, 10 µg/mL, 100 µg/mL, 250 µg/mL, 500 µg/mL, and 1000 µg/mL.

Hydrogen Peroxide Scavenging Activity

The hydrogen peroxide scavenging activity was evaluated using a modified method [17]. Extracts at concentrations of 0.1 µg/mL, 1 µg/mL, 10 µg/mL, 100 µg/mL, 250 µg/mL, 500 µg/mL, and 1000 µg/mL were combined with 20 mM H_2O_2 in a 0.1 M phosphate buffer (pH 7.4) and incubated for 10 minutes. The absorbance was measured at 230 nm against a buffer blank. Controls included a reaction without the extract and a plant control that contained only the buffer. The scavenging activity was calculated using a specific equation.

$$\text{Hydrogen Peroxide Scavenging Activity (\%)} = \frac{A_o - A_s}{A_0} * 100\%$$

Where, A_0= Absorbance of control and A_s= Absorbance of sample

The control refers to the test solution that does not contain a sample, while ascorbic acid was used as the positive control.

Ferrous reducing-antioxidant power (FRAP)

The antioxidant capacity of plant extracts was assessed using a modified FRAP assay [18]. Extracts at concentrations of 12.5 to 100 µg/mL were combined with a phosphate buffer (0.2 mM, pH 6.6) and 1% potassium ferricyanide, followed by incubation at 50°C for 20 minutes. After the addition of 10% trichloroacetic acid, the mixture was centrifuged, and the supernatant was combined with distilled water and ferric chloride. The absorbance was measured at 700 nm using a UV-Visible spectrophotometer, with ascorbic acid serving as the standard.

In-vitro Alpha-Amylase Inhibitory Activity of *A. bicolor*

The α-amylase inhibition assay was performed using the 3,5-dinitrosalicylic acid (DNSA) method [19]. A 200 µL solution of α-amylase (5 mg/100 ml) was combined with 200 µL of the plant extract and incubated for 10 minutes at 30°C. Following this, 200 µL of a 1% starch solution was added, and the mixture was incubated for an additional 3 minutes. The reaction was halted by adding 200 µL of DNSA reagent and boiling the mixture for 10 minutes at 85–90°C. After cooling, the solution was diluted with 5 ml of distilled water, and its absorbance was measured at 540 nm using a UV-Visible spectrophotometer. Controls included a blank that represented 100% enzyme activity, a plant control without the enzyme, and a positive control using acarbose at various concentrations (50-1000 µg/mL). The α-amylase inhibitory activity was calculated as the percentage of inhibition, and IC_{50} values were derived from the inhibition curves.

In-vivo activity of *A. bicolor* leaves extract

Experimental Animals, Housing, and Humane endpoint

Healthy Wistar Albino rats (N=35), weighing between 200-250 g, between 8-10 weeek olds rats were sourced from the Animal House Division of the Department of Plant Resources in Kathmandu, Nepal. We used male rats for the analgesis and antiinflammatory activity, whereas female rats were used for acute toxicity study. They were kept in polypropylene cages within the primate facility at Pokhara University, where conditions were maintained at room temperature (25±3°C, 55±5% humidity) with a natural light-dark cycle of 12 hours. The rats were allowed to acclimate for two weeks on a normal diet and had access to water ad libitum, following the guidelines set forth by the National Research Council [20]. All experimental animals were randomly assigned the different group with blinding prior to the experiments. Following the completion of experiments, all animals were anesthetized with an intramuscular injection of 3 ml ketamine. Once under anesthesia, the experimental animals were then euthanized through cervical dislocation.

Ethics Statement

The research was conducted in compliance with the National Institutes of Health (NIH) guidelines for the Care and Use of Laboratory Animals. The Institutional Review Committee (IRC) at the Pokhara University Research Center (PURC) on the Ethics of Animal Experiments reviewed and approved the study protocol (Protocol Number: 105/079/80). During all research involving animal subjects was carried out in accordance with the regulations and guidelines established by NIH and every effort was made to reduce animal suffering.

Acute toxicity studies

The acute toxicity of the ethanolic extract of *A. bicolor* was evaluated following OECD guideline 425, utilizing healthy, non-pregnant, nulliparous female rats. The rats underwent an 18-hour fasting period before being administered a single oral dose of 2000 mg/kg body weight [21]. They were observed continuously for 4 hours (at 30-minute intervals) and then daily for 2 weeks for signs of toxicity, including diarrhea, weight loss, lethargy, tremor, paralysis, food and water intake, and mortality.

In-Vivo Analgesic activity

Hot plate Test

The analgesic activity of the ethanolic extract of *A. bicolor* was assessed using the Hot Plate Test in male Wistar albino rats. A total of thirty rats were randomly divided into five groups, which included a control group and a positive control group that received morphine (5 mg/kg, I.P.). The hot plate was set at 55 ± 0.5°C to elicit pain, and the response times (paw licking or jumping) were recorded at intervals of 0, 30, 60, 90, and 120 minutes, with a cutoff time of 15 seconds to avoid injury. The reaction times of the groups treated with the extract were compared to those of the control group [22, 23].

Tail Flick Method

The analgesic activity of the ethanolic extract of *A. bicolor* was evaluated using the Tail Flick method in male Wistar albino rats. A total of thirty rats were divided into five groups, including a control group and a positive control group that received morphine (5 mg/kg, I.P.). To induce pain, 1–2 cm of the rats' tails were immersed in a water bath at 55 ± 0.5°C after administering the extract, standard, or control for 30 minutes. The reaction time, indicated by tail deflection, was recorded at 0, 30, 60, 90, and 120 minutes, with a 15-second cutoff to

prevent injury. The reaction times of the groups treated with the extract were compared to the control group [24].

In the treatment protocols designed to assess analgesic activity, the experimental animals were separated into five groups (n = 6). The control group received 2% Tween-80 (10 ml/kg, orally), while the positive control group was administered morphine (5 mg/kg, I.P.). The test groups received varying doses of *A. bicolor* ethanolic extract: 125 mg/kg (Test I), 250 mg/kg (Test II), and 500 mg/kg (Test III), all given orally.

In-Vivo **Anti-inflammatory Activity of A. bicolor leaves extract**

The anti-inflammatory activity of the ethanolic extract of *A. bicolor* was assessed using the Carrageenan-Induced Paw Edema method, with modifications based on Winter et al. [25]. A total of thirty albino rats were divided into five groups and fasted overnight, with access to water. Inflammation was induced by injecting 0.1 ml of 1% Carrageenan into the right hind paw of each rat. The extracts and diclofenac (as a standard) were administered orally one hour prior to the Carrageenan injection. Paw volume was measured using a digital Vernier caliper before the injection and at 1, 2, 3, and 4 hours post-injection.

In the treatment protocols designed to evaluate anti-inflammatory activity, the control group received 2% Tween-80 (10 ml/kg, orally). The positive control group was administered diclofenac (50 mg/kg, p.o.). The test groups were given *A. bicolor* ethanolic extract at doses of 125 mg/kg (Test I), 250 mg/kg (Test II), and 500 mg/kg (Test III), all via oral administration. The percentage of edema inhibition was calculated as a percentage of the difference in the inflammation index (Pi) using the following formula:

% Edema inhibition = $(C_0-T_0/C_0)*100$

Where C_0 refers to the control group, and T_0 denotes the test group.

Inflammation Index $(P_i) = D_t - D_0$

Where, D_t represents the final diameter of the paw that was injected with carrageenan, and D_0 denotes the initial diameter of the same paw prior to the injection of carrageenan.

Results

Phytochemical screening of *A. bicolor* extract

Qualitative phytochemical analysis indicated the presence of various phytoconstituents, including tannins, flavonoids, saponins, carbohydrates, terpenoids, and cardiac glycosides in the methanol extracts, as summarized in Table 1.

Table 1. Phytochemical screening of ethanol extract of *A. bicolor* leaves.

Phytochemical Test		***A. bicolor* extract**
Alkaloid	Wagner test	-
Flavonoid	Alkaline reagent test	+
Phenol	Sodium hydroxide test	+
Tannin	Ferric chloride Test	+
Glycoside	Salkowski's Test	-
Saponin	Lead acetate test	+
Carbohydrate	Fehling test	-
+, Presence and -, absence		

TLC Profiling of *A. bicolor* extracts

TLC profiles of *A. bicolor* extracts are shown in **Error! Reference source not found.**. The chromatogram visualized under a UV chamber revealed separation of several band at 254 nm and 365 nm.Yellow, reddish brown and black colors appears on chromatogram on sulphuric acid spray followed by heat. Ferric chloride spray leads to appearance of separate brown, dark blue and green band. Pale Yellow color band against the violet color background chromatogram were obtained after dipping the developed TLC plate in DPPH solution.

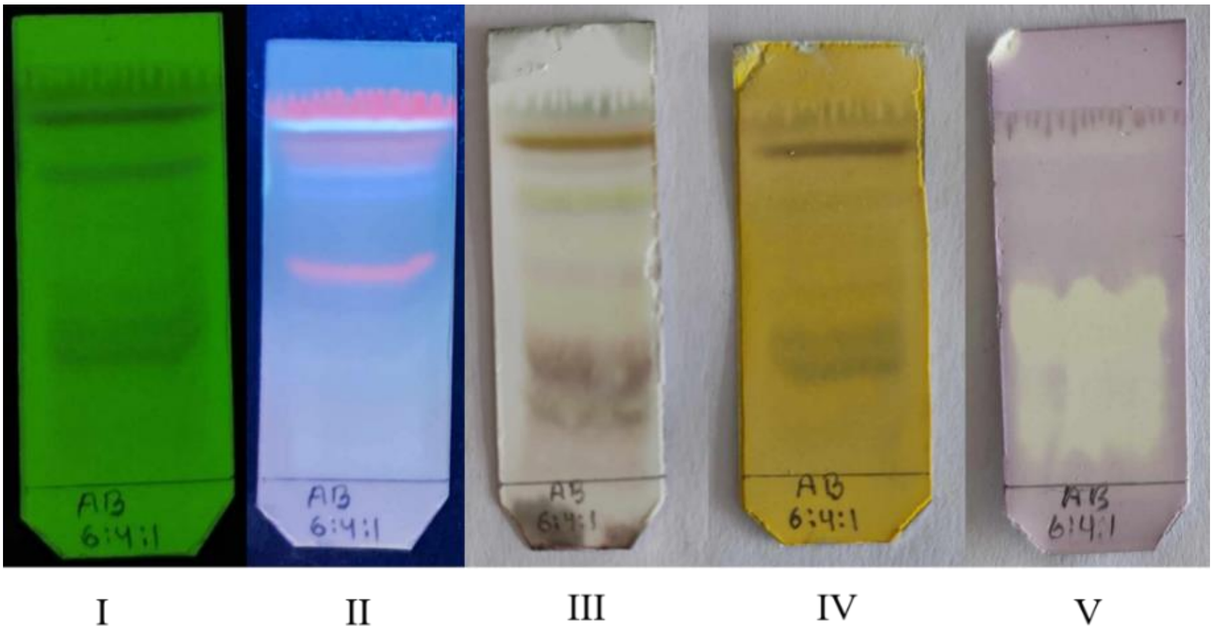

Figure 1. TLC plate observation, I; TLC spot of *A. bicolor* extract observation under short UV 254 nm, II; long UV 365 nm, III; TLC spot sprayed with 10 % H_2SO_4/Heat, (IV) 10% $FeCl_3$, (V) DPPH.

Total phenolic and flavonoid content estimation of *A. bicolor*

The extraction yield percentage was (15.48%) in the *A. bicolor* leaves ethanolic extracts. Phenol content was (20.98 ± 0.20) mg GAE/gm dry extract weight while flavonoid content was found to be (405.95 ± 0.28) mg QE/gm dry extract weight in ethanolic extract of *A. bicolor*.

In vitro Antioxidant Activity by DPPH, NO, H_2O_2, and FRAP Method

The ethanolic extract of *A. bicolor*leaves demonstrated free radical scavenging activity in the DPPH assay, with an IC_{50} value of 9.87 µg/mL, comparable to that of standard ascorbic acid, as presented in the Table 2. Additionally, the NO free radical scavenging activity of the ethanolic extract (IC_{50} = 72.98 µg/mL) was found to be superior when compared to the standard curcumin. The radical scavenging activity of *A. bicolor* (IC_{50} = 249.59 µg/mL) was higher than that of standard ascorbic acid (IC_{50} = 353.96 µg/mL). Moreover, the reducing power of *A. bicolor* was similar to that of standard ascorbic acid.

Table 2. Antioxidant activity of *A. bicolor* ethanolic extract using DPPH, NO, H_2O_2, and FRAP protocols.

S. N	Antioxidant assay	Sample	Concentration (µg/mL)					IC_{50} (µg/mL)
1	DPPH scavenging (%)		**2.5 µg/mL**	**5 µg/mL**	**7.5 µg/mL**	**15 µg/mL**	**30 µg/mL**	
		A. bicolor	9.25±0.6	43.94±1	50.4±1.0	55.17±0.3	69.63±0.3	**9.87**
		Ascorbic acid	22.2±0.5	48.4±2.1	54.5±1.0	61.34±0.3	76.28±2.0	**3.96**
2	NO scavenging (%)		**10 µg/mL**	**100 µg/mL**	**250 µg/mL**	**500 µg/mL**	**1000 µg/mL**	
		A. bicolor	41.43±1.4	54.98±0.2	68.15±0.4	90.14±0.1	97.55±0.05	**72.98**
		Curcumin	33.89±0.7	9.28±0.6	22.11±0.6	37.33±2.9	91.09±1.34	**162.79**
3	H_2O_2 Scavenging (%)		**-**	**100 µg/mL**	**250 µg/mL**	**500 µg/mL**	**1000 µg/mL**	
		A. bicolor	-	41.8±0.1	49.24±0.3	61.64±4.3	80.8±9.7	**249.59**
		Ascorbic acid	-	28.7±0.2	42.12±0.3	61.89±4.4	94.3±1.7	**359.96**
4	FRAP ($Abs_{700\ nm}$)		**12.5 µg/mL**	**25 µg/mL**	**50 µg/mL**	**75 µg/mL**	**100 µg/mL**	
		A. bicolor	0.139±0.005	0.213±0.002	0.343±0.009	0.47±0.008	0.517±0.004	**-**
		Ascorbic acid	0.234±0.007	0.388±0.008	0.545±0.005	0.797±0.006	1.07±0.006	**-**

DPPH: 2,2-diphenyl-1-picrylhydrazyl. NO: Nitric oxide. H_2O_2: Hydrogen peroxide. FRAP: Ferric ion reducing antioxidantpower. Abs: UV absorbance. IC_{50}: Half-maximal inhibitory concentration.All data are expressed as mean ± standard error of the mean (SEM) (n=3).

Alpha-Amylase Inhibitory Assay

Alpha-amylase inhibitory activity of *A. bicolor* (IC_{50} = 780.58) was found to be higher than standard acarbose (Table 3).

Table 3. Alpha-amylase inhibitory activity of selected plant samples.

S. No.	Sample Name	% Alpha-amylase inhibitory activity					IC_{50} Value (µg/mL)
		50 µg/mL	100 µg/mL	250 µg/mL	500 µg/mL	1000 µg/mL	
1	*A. bicolor*	12.26 ± 0.34	24.84 ± 0.15	29.47± 0.34	50.86 ± 0.27	83.08 ± 0.31	780.58
2	Acarbose	16.06 ± 0.49	14.31 ±0.36	21.88±0.5	35.04±0.24	53.75 ± 1.39	511.51

All data are expressed as mean ± standard error of the mean (SEM) (n=3)

Oral Acute Toxicity Study

The oral administration of the ethanolic extract of *A. bicolor*leaves did not result in any noticeable behavioral changes, including locomotion, activity levels, hair texture, pupil size, or feeding patterns. Furthermore, there were no signs of morbidity or mortality observed at the dose of 5000 mg/kg. Therefore, the LD_{50} of the ethanolic extract is estimated to be greater than 5000 mg/kg, classifying it as Category 5 under the Globally Harmonized System (GHS).

In Vivo Analgesic Activity

Hot Plate Method

The maximum reaction time was observed for the group *A. bicolor* 250 mg/kg at 120 minute (9.77±0.84) as shown in Figure 2 and maximum analgesic activity was observed for group *A. bicolor* 500mg/kg at 60 minute (45.26± 0.95 %) as shown in Table 4, Figure 2.

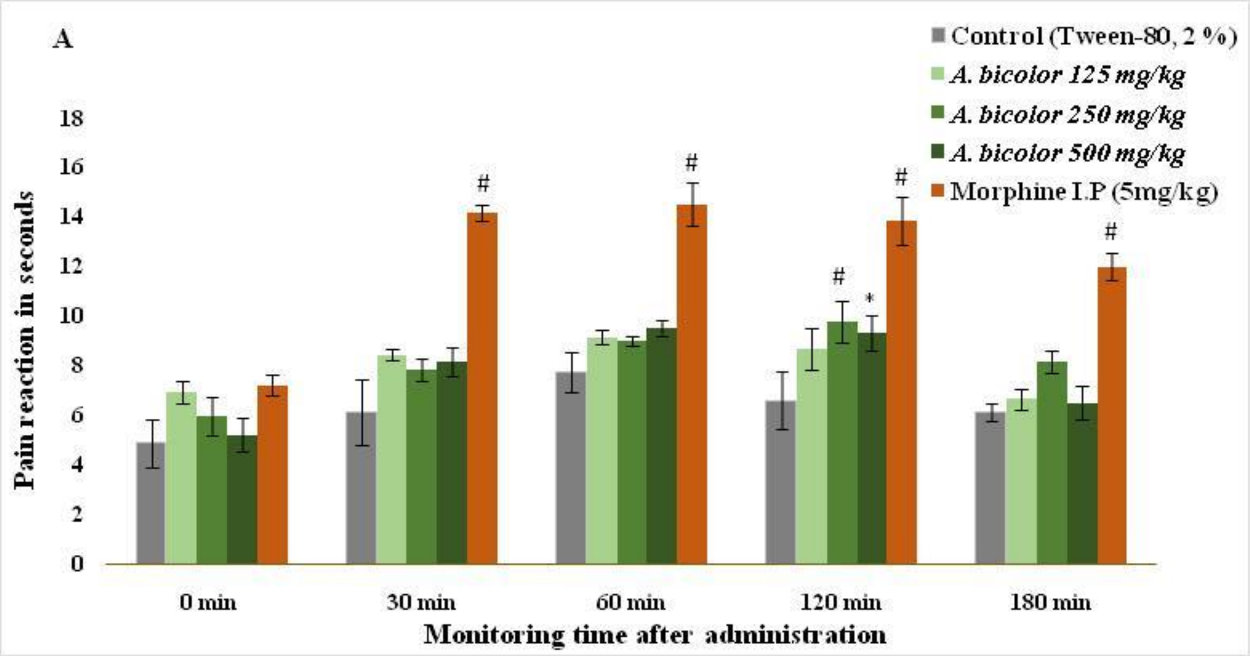

Figure 2. Evaluation of analgesic activity in rat using the hot plate(**A**). Pain reaction time (paw licking or tail withdrawal latency) expressed as mean ± standard error, n = 6 rats per group, Data differed significantly at *p < 0.05, $^{\#}$p<0.001 when compared with the control group in the relevant column using one-way ANOVA with post-hoc Tukey HSD test.

Table 4. Percentage analgesic activity of *A. bicolor* extract in various experimental groups via Hot Plate Method.

**Groups (n=6)**	**Percentage analgesic activity**			
	**30 min**	**60 min**	**120 min**	**180 min**
Morphine I.P.	49.08±0.65**	50.27±0.59**	47.86±0.94**	39.91±0.91**
A. bicolor 125 mg/kg	17.81±0.81**	24.45±0.61**	20.09±1.65**	3.90±0.55**
A. bicolor 250 mg/kg	23.75±0.95**	33.66±0.69**	38.89±0.67**	26.83±0.75**
A. bicolor 500 mg/kg	36.27±0.55	45.26±0.95	44.26±0.38	20±0.49

Data are expressed as mean ± SEM, *p<0.05, **p<0.001 when compared to control.

Tail Flick Method

The maximum reaction time was observed for the group *A. bicolor* 250 mg/kg at 60 minute (7.45±0.62) as shown in Figure 3 and maximum analgesic activity was observed for group *A.*

bicolor 500mg/kg at 120 minute (34.63±2.31 %) as shown in Table 5 and Figure 3.

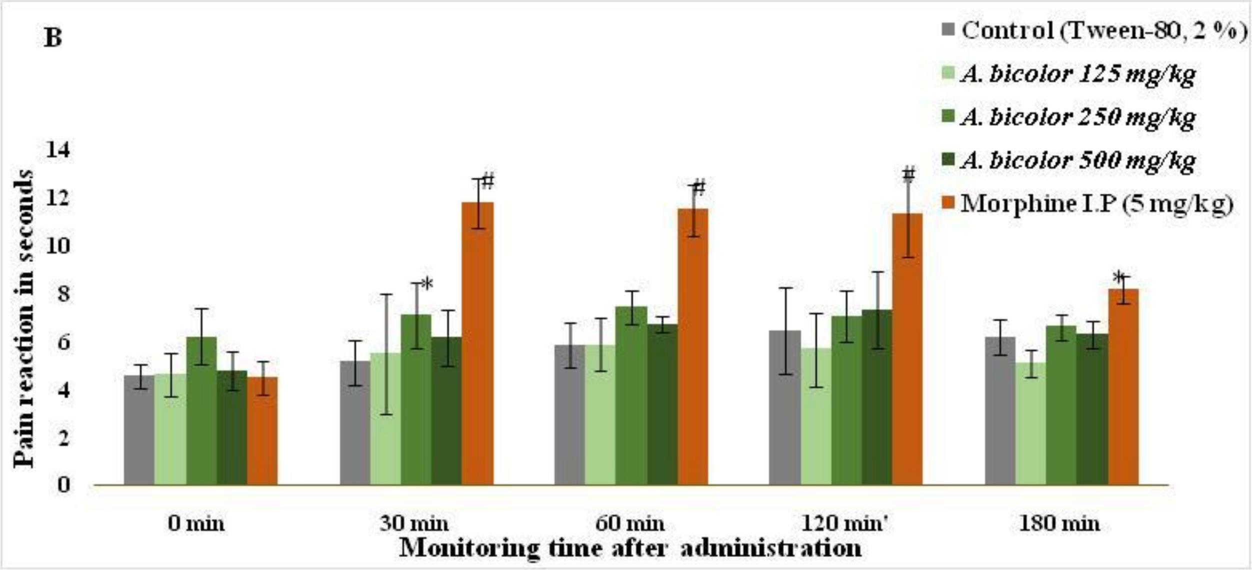

Figure 3. Evaluation of analgesic activity in rat using tail flick test (**B**). Pain reaction time (paw licking or tail withdrawal latency) expressed as mean ± standard error, n = 6 rats per group, Data differed significantly at $*p < 0.05$, $^{\#}p<0.001$ when compared with the control group in the relevant column using one-way ANOVA with post-hoc Tukey HSD test.

Table 5. Percentage analgesic activity of *A. bicolor* extract in various experimental groups via Tail Flick Method.

Group (n=6)	Percentage analgesic activity			
	**30 min**	**60 min**	**120 min**	**180 min**
Morphine I.P	61.81±2.91**	60.93± 1.32**	60.37±2.89	45.07±2.66
A. bicolor 125mg/kg	15.84±0.86	21.16±1.69	18.51±1.51	9.16±0.71
A. bicolor 250mg/kg	12.74±0.53	16.84±0.91	12.06±0.55	6.27±0.29
A. bicolor 500mg/kg	22.19±1.21	28.84±1.89	34.63±2.31	24.04±0.86

Data are expressed as mean ± SEM, *p<0.05, **p<0.001when compared to control

In-Vivo Anti-Inflammatory Activity Carrageenan Induced Paw Edema

Maximum inhibition of paw edema was observed for *A. bicolor* 500 mg/kg extract group (36.44±1.85 %) at three hour after treatment Figure 4.

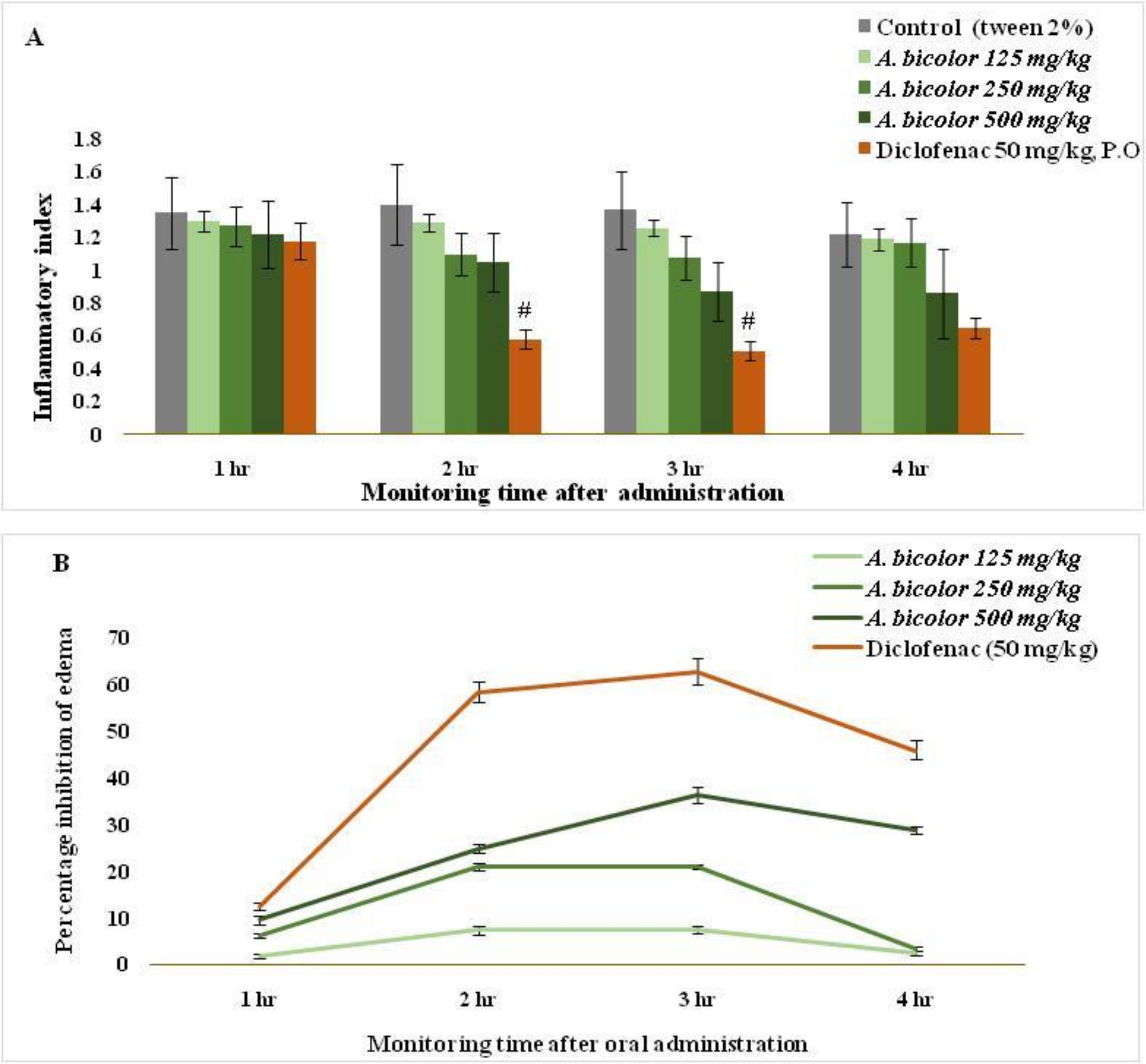

Figure 4. Evaluation of anti-inflammatory activity implementing the carrageenan- induced paw edema model. (**A**): Inflammatory index (edema volume in ml) and (**B**): Percentage inhibition of paw edema (%) expressed as mean ± standard error, n = 6 rats per group. Data differed significantly at *$p < 0.05$, $^{\#}p<0.001$ when compared with the control group in the relevant column using one-way ANOVA with post-hoc Tukey HSD test.

Discussion

Medicinal plants have been increasingly recognized as valuable alternatives for managing various diseases, owing to their affordability, wide availability, and relatively low risk of adverse effects. However, despite of the wide usage of herbal plants for the cure of disease

conditions, the efficacy data on the use of many of these plants are not available [26].

Secondary metabolites from the plants serve an essential purpose in the defense mechanism of plants itself against microorganisms, preys, stress and interspecies protection. The presence of these secondary metabolites in plants is instrumental in predicting their potential effects when consumed as food or used as medicine[27]. Compounds such as saponins, flavonoids, tannins, terpenoids, steroids, and alkaloids contribute to the anti-diabetic, anti-diarrheal, anti-inflammatory, anti-ulcer, anticancer, nephroprotective, and hepatoprotective properties of herbal formulations [3, 28] whereas cardiac glycosides account for the cardiac and neurotropic activities of these herbal preparations.

In this study, the total phenolic and flavonoid content, along with the antioxidant, anti-inflammatory, and analgesic activities of the ethanolic extracts from *A. bicolor* leaves, were evaluated. Phytochemical analysis confirmed the presence of phenols, flavonoids, tannins, and saponins, consistent with findings from previous studies [29]. Additionally, to further validate the phytochemical results, TLC profiling was conducted and compared with earlier research [30], revealing a mixture of compounds in the plant extract. The presence of saccharides is represented by the appearance of black spot observed in the TLC plate. In our study *A. bicolor* extract showed similar black spots confirming the presence of saccharides.

A. bicolor leaves showed brownish grey and yellow spots, similar spots observed in TLC profiling and confirming presence of phenol and flavonoids. Similarly, presence of white spots confirmed the presence potent antioxidant compound in the ethanolic extract. The results was similar with the study conducted by authors [3 1] .

In this study, IC_{50} values for free radical scavenging activities were determined using linear regression from plots of percent radical scavenging activity against the concentration of the tested compounds. All plant extracts demonstrated significant free radical scavenging activity, with the leaf extract of *A. bicolor* exhibiting the highest DPPH scavenging activity,

having an IC_{50} value of 9.87 µg/mL, which closely approximates the value of standard ascorbic acid (IC_{50} 3.96 µg/mL), as shown in Table 2. DPPH assay performed by Tiwari et al. on *A. bicolor* leaf extract reported a scavenging activity with an IC_{50} of 46.76 µg/mL, which is higher than the value observed in this study [29]. Researcher evaluated DPPH scavenging activity on ethanolic extract of similar fern species *Cheilanthes albormarginata* (IC_{50} value 16.33) which was near to our study [32].

Nitric oxide (NO) is a significant bio-regulatory compound with various physiological impacts. These effects encompass the regulation of blood pressure, transmission of neural signals, platelet function, as well as antimicrobial and antitumor activities. During infections and inflammatory conditions, the production of nitric oxide (NO) increases, which can lead to harmful and undesirable effects? The impaired NO release is also believed to induce diabetes which can be prevented by antioxidants. Nitric oxide radicals are formed during the reaction of endogenous nitric oxide when reacts with oxygen or superoxides like NO_2 or N_2O_2 which are very reactive [33]. Nitric oxide (NO) and reactive species are known to disrupt the structural and functional integrity of various cellular components. Plant antioxidants can help mitigate or prevent the harmful chain reactions triggered by excessive NO production, which poses significant health risks. Elevated NO levels are linked to several diseases, including inflammation, cancer, neurodegenerative disorders, and other pathological conditions [34]. The ethanolic extract of *A. bicolor* exhibited strong NO scavenging activity, with an IC_{50} value of 72.98 µg/mL, surpassing the standard antioxidant curcumin, which had an IC_{50} value of 162.79 µg/mL. Notably, this is the first time NO scavenging activity has been evaluated for *A. bicolor*. A study by researcher's on a related species, *Chenopodium alborgiminata*, showed similar results to those observed in this study [32].

Hydrogen peroxide occurs naturally at low concentrations in the air, water, plants, microorganisms, the human body, and food [35]. While it is not inherently reactive, hydrogen

peroxide can become toxic to cells, as it may lead to the formation of hydroxyl radicals (OH•) upon decomposition into oxygen and water [36]. In this study, the hydrogen peroxide assay demonstrated that the ethanolic extract of *A. bicolor*leaves effectively removes hydroxyl radicals, with an IC_{50} value of 249.59 µg/mL, showing stronger hydroxyl radical scavenging activity compared to the standard antioxidant ascorbic acid (IC_{50} 353.96 µg/mL), as presented in Table 2. To the best of our knowledge, this is the first study to evaluate the hydrogen peroxide scavenging activity of *A. bicolor* extract.

The reducing capacity of a sample is considered an important marker of its antioxidant potential. The reducing power values for *A. bicolor* are shown in Table 2. The findings from the reducing power assay followed a similar pattern to those observed in other radical scavenging assays. *A. bicolor* extract exhibited strong reducing capacity when compared to ascorbic acid. These results suggest that the *A. bicolor* extract could serve as an electron donor, interacting with free radicals by converting them into more stable compounds and thereby halting the radical chain reaction [37].

Alpha amylase is a protein that hydrolyses alpha- linked polysaccharide such as starch and glycogen and glycogen and converts them into simple sugars such as glucose and maltose[38]. The alpha-amylase inhibitory activity of *A. bicolor* was found to be moderate (IC_{50} value 780.58 µg/mL) compared to standard drug acarbose (IC_{50} value 511.51 µg/mL).The result is also similar to research carried out by researcher where IC_{50} of *A. bicolor* was found to be $651.58 \pm 10.32\ \mu$g/mL [39].

The in vivo analgesic activity of *A. bicolor* extract was assessed using the hot plate and tail flick tests. The analgesic properties of natural products, particularly plant extracts, have garnered considerable interest due to their potential therapeutic effects and lower side effects compared to synthetic analgesics [40]. Traditionally, *A. bicolor* has been utilized in folk medicine for its analgesic properties [41]. The analgesic effects of *A. bicolor* were evaluated

across various doses of the extract (125 mg/kg, 250 mg/kg, and 500 mg/kg), demonstrating a dose-dependent analgesic response. The analgesic activities for the different doses of *A. bicolor* in the hot plate and tail flick tests are presented in Table 4 and Table 5. The 500 mg/kg dose exhibited analgesic activities of 45.26% and 44.26% at 60 minutes and 120 minutes in the hot plate test, respectively, compared to morphine I.P. In the tail flick test, this dose showed 28.84% and 34.63% analgesic activity at 60 minutes and 120 minutes, respectively, compared to morphine I.P. Similarly, the 250 mg/kg and 125 mg/kg doses of *A. bicolor* showed highly significant activity ($p<0.001$) compared to the control group in the hot plate test. The 250 mg/kg dose also demonstrated significant activity ($p<0.05$) compared to the control in the tail flick test, and this dose showed highly significant activity ($p<0.001$) when compared to the standard morphine in the tail flick method. From both tests, it can be concluded that the moderate dose (250 mg/kg) was adequate to elicit analgesic activity. The analgesic effect of *A. bicolor* may be attributed to its high flavonoid and antioxidant content, as suggested by previous study [42].

Inflammation often plays a critical role in the pathological progression of organ diseases. The three primary pathways involved in inflammation—NF-κB, MAPK, and JAK-STAT—are crucial, and any dysregulation of one or more of these pathways can contribute to inflammation-related diseases [43]. This underscores the importance of developing new, safe, and effective agents to mitigate the effects of inflammation. Inflammation can impact various organs, including the heart, pancreas, liver, kidneys, lungs, brain, intestinal tract, and reproductive system, potentially leading to tissue damage or disease. Therefore, it is essential to screen for anti-inflammatory phytochemicals that may help slow the progression of chronic diseases. One such chronic condition associated with inflammatory responses in the body is diabetes [43].

The carrageenan-induced acute inflammation model is widely recognized as the most effective method for assessing the efficacy of anti-inflammatory agents. In this model, the onset of edema in the hind paw of rats is typically represented by a biphasic curve. The initial phase of inflammation occurs within an hour after the administration of carrageenan, primarily due to trauma at the injection site and the involvement of serotonin and histamine [44].

The in vivo anti-inflammatory activity of the ethanolic extract of *A. bicolor*leaves was evaluated in normal rats, focusing on its antioxidant activity, total phenolic content, and flavonoid content. The study revealed a significant anti-inflammatory effect of the *A. bicolor* extract, which moderately reduced paw edema volume at all doses between 3 to 4 hours post-administration, compared to the control group. The most considerable reduction in edema, estimated at 36.44% ($p<0.05$), occurred at the 500 mg/kg dose, specifically at 3 hours after carrageenan injection. Notably, the extract did not affect the first phase of edema (1 to 2 hours), indicating that it did not inhibit histamine and serotonin during this initial phase. However, during the second phase, the extract demonstrated effective anti-inflammatory activity. Similar findings were reported by Shaikh et al., who noted that medicinal plants targeting the COX pathway significantly reduced edema volume at the 3-hour mark [45].

Flavonoids, stilbenes, and alkaloids are recognized as key natural compounds with anti-inflammatory properties [46]. In our study, the flavonoid content in *A. bicolor* extract was notably high (405.95 ± 0.28 mg QE/g). The anti-inflammatory activity may be attributed to this high flavonoid content, as flavonoids exert their effects through various mechanisms, including the potential to reduce neutrophil degranulation [47]. Additionally, previous studies have highlighted the anti-inflammatory roles of phytochemicals such as phenols, polysaccharides, terpenoids, saponins, and alkaloids [48]. Our findings also indicated the presence of many of these phytochemicals, which may contribute to the observed reduction in

paw edema volume. However, further studies at the molecular level are recommended to explore the underlying mechanisms.

Conclusion

In conclusion, *A. bicolor* demonstrates considerable promise as a source of bioactive compounds with notable antioxidant, analgesic, anti-inflammatory, and hypoglycemic activities. Phytochemical screening of its ethanolic extract revealed the presence of alkaloids, flavonoids, phenols, tannins, and carbohydrates, with particularly high levels of flavonoids. The extract exhibited strong antioxidant activity, as evidenced by its DPPH scavenging ability (IC_{50} value 9.87 µg/mL), while its alpha-amylase inhibitory activity (IC_{50} = 780.58 µg/mL) highlights its potential as a hypoglycemic agent. In vivo experiments further confirmed the analgesic and anti-inflammatory effects, likely attributed to its high flavonoid and antioxidant content. These findings underscore the necessity of further research to isolate and characterize the active constituents and investigate their mechanisms of action, paving the way for the development of novel analgesic, anti-inflammatory, and antidiabetic therapies derived from *A. bicolor*.

Data Availability Statement

All relevant data is in the paper and if any query regarding the finding of this study is obtainable from the corresponding author upon request.

Competing interests

The authors have declared that no competing interests exist.

Funding

The author(s) received no specific funding for this work.

Acknowledgments

The authors express their gratitude to Pokhara University, Kaski, Nepal's Department of Pharmaceutical Sciences, School of Health and Allied Sciences for providing the research facilities and the laboratory facilities throughout the research. Sincere thanks are also extended to the supervisor Associate Professor Dr. Sushil Panta.

Abbreviations

DMSO, Dimethyl sulfoxide, DPPH, 2,2-diphenyl-1-picrylhydrazyl; NO, Nitric oxide; H_2O_2, Hydrogen peroxide; SEM, Standard Error Mean: *A. bicolor, Aleuritopteris bicolor.*

Author contributions

Conceived and designed the experiments: PKJ, SP2.

Performed the experiments: PKJ, RKY, SKC, SP1, BP.

Data analyzed and analysis tool: PKJ, BP.

Writing of the manuscript: PKJ, SP2, RKY, SKC, BP.

Supervision: SP2.

Revised manuscript critically for important intellectual content: PKJ, BP, SP2.

Reference

1. Gusev E, Zhuravleva Y. Inflammation: A new look at an old problem. International journal of molecular sciences. 2022;23(9):4596.
2. Navarrete S, Solar C, Tapia R, Pereira J, Fuentes E, Palomo I. Pathophysiology of deep vein thrombosis. Clinical and experimental medicine. 2023;23(3):645-54.
3. Pandey B, Baral R, Kaundinnyayana A, Panta S. Promising hepatoprotective agents from the natural sources: a study of scientific evidence. Egyptian Liver Journal. 2023;13(1):14.
4. Rouadi PW, Idriss SA, Naclerio RM, Peden DB, Ansotegui IJ, Canonica GW, et al. Immunopathological features of air pollution and its impact on inflammatory airway diseases (IAD). World Allergy Organization Journal. 2020;13(10):100467.
5. Machado GC, Abdel-Shaheed C, Underwood M, Day RO. Non-steroidal anti-inflammatory drugs (NSAIDs) for musculoskeletal pain. Bmj. 2021;372.
6. Muhammad N, Haq IU, Jan MS, AlOmar TS, Rauf A, Wadood A, et al. In-vitro and in-vivo assessment of the anti-diabetic, analgesic, and anti-inflammatory potenstials of metal-based carboxylates derivative. Heliyon. 2023;9(8).
7. Shahrestanaki E, Mohammadian Khonsari N, Seif E, Baygi F, Ejtahed H-S, Sheidaei A, et al. The worldwide trend in diabetes awareness, treatment, and control from 1985 to 2022: a systematic review and meta-analysis of 233 population-representative studies. Frontiers in Public Health. 2024;12:1305304.
8. MacLeod J, Franz MJ, Handu D, Gradwell E, Brown C, Evert A, et al. Academy of Nutrition and Dietetics Nutrition Practice Guideline for Type 1 and Type 2 Diabetes in Adults: Nutrition Intervention Evidence Reviews and Recommendations. Journal of the Academy of Nutrition and Dietetics. 2017;117(10):1637-58.
9. Dayma V, Chopra J, Sharma P, Dwivedi A, Tripathi IP, Bhargava A, et al. Synthesis, antidiabetic, antioxidant and anti-inflammatory activities of novel hydroxytriazenes based on sulpha drugs. Heliyon. 2020;6(8).
10. Sureshkumar J, Ayyanar M, Silambarasan R. Pteridophyte species richness along elevation gradients in Kolli Hills of the Eastern Ghats, India. Journal of Asia-Pacific Biodiversity. 2020;13(1):92-106.
11. Bhattarai S, Kunwar RM. Nepalese Pteridophytes Used as Antimicrobials: Challenges and Opportunities. Promising Antimicrobials from Natural Products. 2022:15-30.
12. Pandey B, Thapa S, Kaundinnyayana A, Panta S. Hepatoprotective effects of Juglans regia on carbon tetrachloride-induced hepatotoxicity: In silico/in vivo approach. Food Science & Nutrition. 2024.
13. Shaikh JR, Patil M. Qualitative tests for preliminary phytochemical screening: An overview. International Journal of Chemical Studies. 2020;8(2):603-8.
14. Aryal S, Baniya MK, Danekhu K, Kunwar P, Gurung R, Koirala N. Total phenolic content, flavonoid content and antioxidant potential of wild vegetables from Western Nepal. Plants. 2019;8(4):96.
15. Losada-Barreiro S, Sova M, Mravljak J, Saso L, Bravo-Díaz C. Synthesis, in vitro antioxidant properties and distribution of a new cyanothiophene-based phenolic compound in olive oil-in-water emulsions. Antioxidants. 2020;9(7):623.
16. Parul R, Kundu SK, Saha P. In vitro nitric oxide scavenging activity of methanol extracts of three Bangladeshi medicinal plants. Pharma Innov. 2013;1(12):83-8.

17. Al-Amiery AA, Al-Majedy YK, Kadhum AAH, Mohamad AB. Hydrogen peroxide scavenging activity of novel coumarins synthesized using different approaches. PLoS ONE. 2015;10(7):e0132175.
18. Oh J, Jo H, Cho AR, Kim S-J, Han J. Antioxidant and antimicrobial activities of various leafy herbal teas. Food control. 2013;31(2):403-9.
19. Miller GL. Use of dinitrosalicylic acid reagent for determination of reducing sugar. Analytical chemistry. 1959;31(3):426-8.
20. Care IoLARCo, Animals UoL. Guide for the care and use of laboratory animals: US Department of Health and Human Services, Public Health Service, National …; 1986.
21. Chemicals D. OECD Guideline for testing of chemicals. The Organisation for Economic Co-operation and Development: Paris, France. 2005:1-13.
22. Eddy NB, Leimbach D. Synthetic analgesics. II. Dithienylbutenyl-and dithienylbutylamines. Journal of Pharmacology and Experimental Therapeutics. 1953;107(3):385-93.
23. KC S, Pandey B, KC S, Gurung S, Gautam A. Anti-inflammatory, analgesic, and acute toxicity evaluation of Litchi chinensis seed extract in albino rat. Natural Resources for Human Health. 2021;1(1):30-5.
24. Hasan MM, Uddin N, Hasan MR, Islam AM, Hossain MM, Rahman AB, et al. Analgesic and Anti-Inflammatory Activities of Leaf Extract of Mallotus repandus (Willd.) Muell. Arg. BioMed Research International. 2014;2014(1):539807.
25. Winter CA, Risley EA, Nuss GW. Carrageenin-induced edema in hind paw of the rat as an assay for antiinflammatory drugs. Proceedings of the society for experimental biology and medicine. 1962;111(3):544-7.
26. Süntar I. Importance of ethnopharmacological studies in drug discovery: role of medicinal plants. Phytochemistry Reviews. 2020;19(5):1199-209.
27. Ramawat KG, Goyal S. Co-evolution of secondary metabolites during biological competition for survival and advantage: An overview. Co-evolution of secondary metabolites. 2020:3-17.
28. Koche D, Shirsat R, Kawale M. An overerview of major classes of phytochemicals: their types and role in disease prevention. Hislopia J. 2016;9(1/2):1-11.
29. Tiwari R, Baral R, Parajuli N, Shrestha R, Pun S, Pahari A, et al. Phytochemical screening, free radical scavenging and In-vitro anti-bacterial activity of ethanolic extracts of selected medicinal plants of Nepal and effort towards formulation of antibacterial cream from the extracts. International Journal of Herbal Medicine. 2021;9(3):39-47.
30. Sonam M, Singh RP, Pooja S. Phytochemical screening and TLC profiling of various extracts of Reinwardtia indica. International Journal of Pharmacognosy and Phytochemical Research. 2017;9(4):523-7.
31. Giri S, Giri U, Subedi K, Magar KT, Pant S, Joshi KR. Thin layer chromatography (TLC) based chemical profiling and antioxidant activity of selected Nepalese medicinal plants. Journal of Health and Allied Sciences. 2020;10(2):15-22.
32. Lamichhane R, Kim S-G, Poudel A, Sharma D, Lee K-H, Jung H-J. Evaluation of in vitro and in vivo biological activities of Cheilanthes albomarginata Clarke. BMC complementary and alternative medicine. 2014;14:1-11.
33. Lundberg JO, Weitzberg E. Nitric oxide signaling in health and disease. Cell. 2022;185(16):2853-78.

34. Lisi F, Zelikin AN, Chandrawati R. Nitric oxide to fight viral infections. Advanced Science. 2021;8(7):2003895.
35. Durmaz L. Antioxidant, antiepileptic, and anticholinergic properties of 4′, 5, 7-Trihydroxy-3, 6-dimethoxyflavone as natural phenolic compound: a toxicology approach. Toxin reviews. 2021;40(3):292-9.
36. Munir S. Sources of ROS Species an Its Harmful and Benefical Effects on Human Health. 2021.
37. Sethi S, Prakash O, Kumar R, Dubey SK, Arya M, Pant AK. Phytochemical Analysis, Antioxidant and Antifungal Activity of Essential oil and Extracts of Alpinia malaccensis (Burm. f.) Roscoe flowers. Brazilian Journal of Pharmaceutical Sciences. 2023;58:e201209.
38. Ćorković I, Gašo-Sokač D, Pichler A, Šimunović J, Kopjar M. Dietary polyphenols as natural inhibitors of α-amylase and α-glucosidase. Life. 2022;12(11):1692.
39. Pandey LK, Sharma KR. Analysis of Phenolic and Flavonoid Content, α-Amylase Inhibitory and Free Radical Scavenging Activities of Some Medicinal Plants. The Scientific World Journal. 2022;2022(1):4000707.
40. Kopustinskiene DM, Bernatonyte U, Maslii Y, Herbina N, Bernatoniene J. Natural herbal non-opioid topical pain relievers—comparison with traditional therapy. Pharmaceutics. 2022;14(12):2648.
41. Manandhar NP. Plants and people of Nepal: Timber press; 2002.
42. Xiao X, Wang X, Gui X, Chen L, Huang B. Natural flavonoids as promising analgesic candidates: a systematic review. Chemistry & biodiversity. 2016;13(11):1427-40.
43. Davis BK, Wen H, Ting JP-Y. The inflammasome NLRs in immunity, inflammation, and associated diseases. Annual review of immunology. 2011;29(1):707-35.
44. Crunkhorn P, Meacock S. Mediators of the inflammation induced in the rat paw by carrageenin. British journal of pharmacology. 1971;42(3):392-402.
45. Shaikh RU, Pund MM, Gacche RN. Evaluation of anti-inflammatory activity of selected medicinal plants used in Indian traditional medication system in vitro as well as in vivo. Journal of Traditional and Complementary Medicine. 2016;6(4):355-61.
46. Kong M, Xie K, Lv M, Li J, Yao J, Yan K, et al. Anti-inflammatory phytochemicals for the treatment of diabetes and its complications: Lessons learned and future promise. Biomedicine & Pharmacotherapy. 2021;133:110975.
47. Hoult J, Moroney MA, Payá M. [44] Actions of flavonoids and coumarins on lipoxygenase and cyclooxygenase. Methods in enzymology: Elsevier; 1994. p. 443-54.
48. Zhu F, Du B, Xu B. Anti-inflammatory effects of phytochemicals from fruits, vegetables, and food legumes: A review. Critical Reviews in Food Science and Nutrition. 2018;58(8):1260-70.

Graphical Abstract

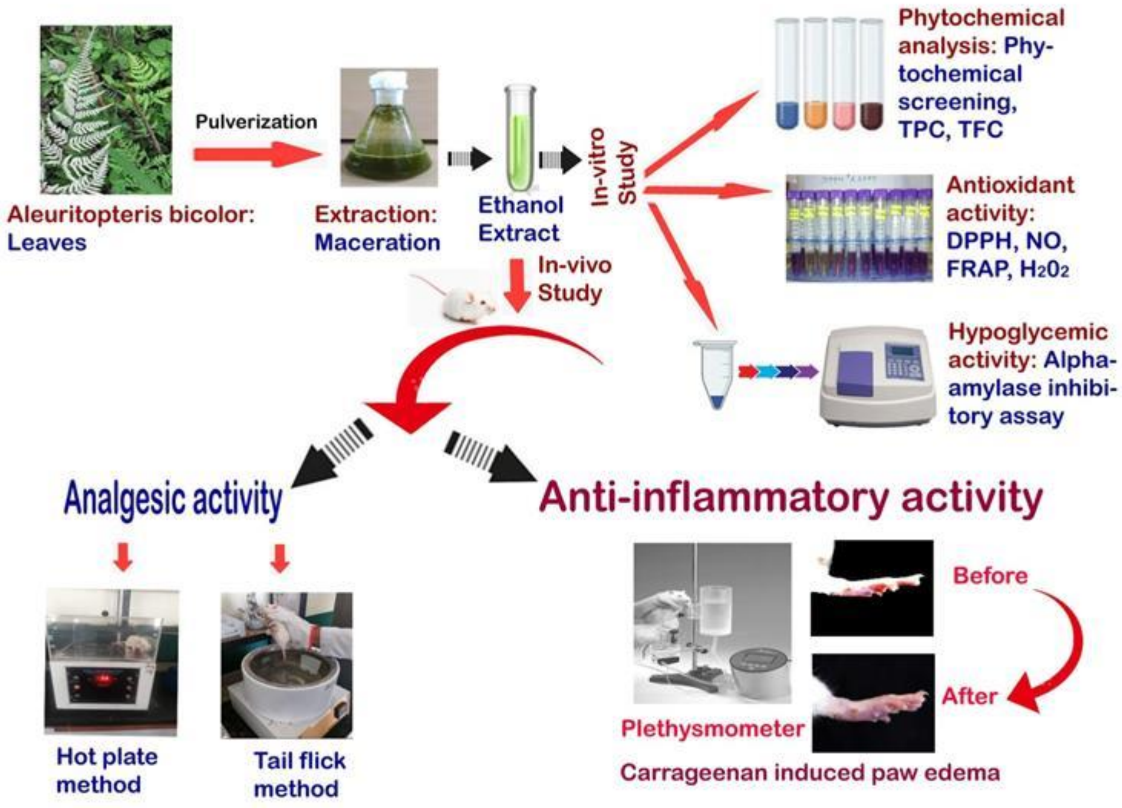

Manuscript Highlights

- This study is the first to report the anti-inflammatory, analgesic, and alpha-amylase inhibitory activities of *Aleuritopteris bicolor* leaves, traditionally used in Nepal.
- The extract exhibited potent antioxidant activity and demonstrated significant analgesic (38.89%) and anti-inflammatory (36.44%) effects in vivo, along with strong alpha-amylase inhibition (IC50: 59.31 µg/mL), highlighting its potential for hypoglycemic therapy.
- These novel findings underscore *A. bicolor* as a promising source for developing natural anti-inflammatory, analgesic, and antidiabetic agents.
