# Supplementary material for: Anti-inflammatory, analgesic, antioxidant, and alpha-amylase inhibitory effects of the hydroethanolic leaf extract of Aleuritopteris bicolor (Roxb.) Fraser-Jenk
Source: PLoS One. 2025 Jun 23;20(6):e0326808. doi: 10.1371/journal.pone.0326808 (PMC12184904; doi:10.1371/journal.pone.0326808)
Supplement: Attachment — Submitted filename: 2nd-Response to Reviewers.docx [file pone.0326808.s004.docx]

**18 February, 2025**

**Respected Academic Editor and Reviewer,**

**Greetings of the day!**

**Thank you for your time and valuable suggestions on our Manuscript (ID:** PONE-D-24-47035R1**) entitled "**Phytochemical Analysis and Anti-Inflammatory, Analgesic, Alpha-amylase, Antioxidant Activities of Ethanolic Extract of *Aleuritopteris bicolor* Leaves Grown in Nepal**". We have addressed the reviewer comment and incorporated in the manuscript. We have submitted two separate files of manuscript.**

**1. In the first file, all the revised sentences are highlighted with red color/track changes to show proof of the revision.**

**2. The second file is also the revised file, but the revised sections are not highlighted, and the file is clean here for further process.**

**3. The third file contains the final figure file for the further process.**

**All the revision as requested by the reviewer was addressed and point-to-point answers are given in the response file for each reviewer's questions and comments. We have also corrected the manuscript as per the journal guidelines and also adding the DOI and PMID id in the references file in the manuscript. We believe that the revised version of the manuscript has significantly improved for the publication.**

**I humbly request you to kindly inform me, if you need any further corrections from our side in the future.**

**Thank You.**

**Best Regards,**

**Bipindra pandey (Corresponding author)**

**Email:** [bipindra.p101@gmail.com](mailto:bipindra.p101@gmail.com)

**RESPONSE TO REVIEWER:**

**Reviewer #3:** The article provides thorough and well-described results, which could interest the wide audience of PLOS One. The authors seem to have addressed most of the comments by previous reviewers, but not all: the title of the manuscript could be more concise, and the Introduction still needs, in my opinion, substantial rewriting. Moreover, as a new reviewer, there are also a few other comments I’d like to make:

**General comment:**
- The writing of the manuscript should be refined. A few English/grammar and spelling mistakes need to be corrected

*Response:* Thank you for your insightful feedback and gone through the whole manuscript and try to correcting grammar, and spelling mistake which was shown in track changes files.


**Title:**
- It's not necessary to emphasize, in the title, every aspect covered in the study. Since phytochemical analysis can always be expected in articles investigating the pharmacological activity of plant extracts, and the results provided by it were not the strongest results of the study, this part could be omitted from the title.

*Response:*

- Thank you for your insightful feedback regarding the title of our manuscript. We appreciate your suggestion to streamline the title by omitting the mention of phytochemical analysis, given its expected presence in studies on the pharmacological activity of plant extracts.

In response, we have revised the title accordingly " Pharmacological Activity of *Aleuritopteris bicolor*: Anti-inflammatory, Analgesic, Antioxidant, and Alpha-amylase Inhibitory Properties" to enhance clarity and focus on the study’s strongest findings.

**Abstract:**
- There is no need to introduce “A. bicolor” as an abbreviation of “Aleuritopteris bicolor,” as in “Aleuritopteris bicolor (A. bicolor)...”.
- The introductory sentence of the abstract states that A. bicolor is “ traditionally used for treating stomach discomfort and aiding wound healing”. In the second sentence, the authors state the aim of the study, which does not have to do with investigating the plant for stomach discomfort or wound healing. Therefore, it would be more coherent if the authors started the abstract with another, more general sentence, like “A. bicolor is used in the traditional medicine of Nepal for a variety of ailments”, for example.

*Response:*

- Thank you for your valuable suggestion. We have removed the unnecessary abbreviation and retained the full botanical name Aleuritopteris bicolor as per your recommendation.
- Thank you for your insightful suggestion. We have revised the introductory sentence of the abstract to provide a more general context regarding the traditional use of Aleuritopteris bicolor, ensuring coherence with the study’s aim by adding general line “*Aleuritopteris bicolor* is used in the traditional medicine of Nepal for a variety of ailments.

**Introduction:**
- Links between information are lacking within and between paragraphs; many sentences feel loosely tied to one another. For example, in the Introduction's first paragraph (which is huge), inflammation, pain, and diabetes are defined in sequence and provided mini-backgrounds, but these conditions are never linked with one another, even though they definitely could. This makes it hard to understand the literature gap(s) the study is supposed to fill.
- In the sentence “This leads to increased vascular permeability and enhanced blood flow, resulting in congestion and thrombosis,” substitute “resulting” with “which can result.”
- It is said that NSAIDs “[i]nitially… were regarded as safe, but subsequent literature reports have revealed adverse reactions.” I understand what the authors meant, but this sentence implies that displaying adverse effects equates to being “unsafe,” which is not appropriate in pharmacology. Please, reformulate.

*Response:*

- Thank you for your insightful feedback. We have revised the Introduction to improve coherence by establishing clearer connections between inflammation, pain, and diabetes, highlighting their interrelationships. Additionally, we have refined the paragraph structure to better articulate the literature gap the study aims to address by adding the following points:

"Current diabetes treatments such as oral hypoglycemic agents and insulin have shortcomings, including hypoglycemia, increased body mass, and additional complications, underscoring the need for new antidiabetic targets and glycemic control strategies. The limitations of existing therapies in managing hyperglycemia without adverse effects, along with their high cost and limited accessibility, inspired to investigate traditional herbal remedies as potential alternatives for diabetes management[9]. "

- Thank you for your valuable feedback. In the sentence “This leads to increased vascular permeability and enhanced blood flow, resulting in congestion and thrombosis,” “resulting” was changed with “which can result.” as per your suggestion.
- Thank you for your valuable feedback. We have revised the sentence to more accurately reflect the pharmacological perspective, ensuring that it does not equate adverse effects with being inherently unsafe as follows:

"Non-steroidal anti-inflammatory drugs (NSAIDs) are effective inhibitors of cyclooxygenase (COX) which is commonly utilized to treat inflammatory diseases and manage pain [5]. In spite of this, some literature reports NSAIDs cause notable side effects such as gastrointestinal discomfort and kidney complications, which are primarily attributed to the free COOH group in their structure [6]. Since for searching the safe and effective alternative drug candidates from the natural products is necessary for treating the inflammation and pain."

**Materials and methods:**
- Some reagents were provided by “Mark, Germany”. I was wondering if “Mark” is not actually “Merck”.
- I suggest the authors clarify they obtained a “concentrated crude extract,” as it is likely that not all ethanol was removed from the extract during rotary evaporation.
- The doses of A. bicolor extract that the animals received when investigating analgesic activity need to be informed along the protocol. The doses were informed in a separate paragraph after other protocol details, in a confusing manner and with some repetition regarding the controls.

*Response:*

- In methodology section “Mark” was corrected as “Merck” as per suggestion.
- Thank you for your suggestion. We have clarified in the manuscript that the extract obtained is a “concentrated crude extract” to accurately reflect the potential presence of residual ethanol after rotary evaporation.
- Thank you for your valuable feedback. We have revised the manuscript to integrate the dosage information directly within the protocol description in both method of analgesic activity for ensuring clarity and eliminating redundancy regarding the controls.

**Results:**
- The sentences “The hydroxyl radical, an exceptionally reactive free radical, is… free radical pathology. This radical is capable of… within living cells” should be in the Introduction or Discussion. Similarly, the classification of A. bicolor extract “as Category 5 under the Globally Harmonized System (GHS)” should be stated in the Discussion, along with the implications of such classification (what does GHS Category 5 cover?).

*Response:*

- The sentences “The hydroxyl radical, an exceptionally reactive free radical, is… free radical pathology. This radical is capable of… within living cells” was placed in the discussion part as per suggestion.

- Similarly the classification of A. bicolor extract “as Category 5 under the Globally Harmonized System (GHS)” was included in the discussion part and written as follows;

LD_50_ of the ethanolic extract of *A. bicolor* extract is estimated to be greater than 5000 mg/kg, classifying it as Category 5 under the Globally Harmonized System of Classification and Labelling of Chemicals (GSH) which indicates that the substance is the least toxic category if swallowed.

**Discussion:**
- The first paragraph of the Discussion is too long for containing only generic information.
- By “A. bicolor leaves showed brownish grey and yellow spots”, did the authors mean that the TLC showed “grey and yellow spots”? Please, clarify.
- Substitute “Cheilanthes albormarginata” with “Aleuritopteris albomarginata” (accepted/current species name).
- Reformulate the paragraph that starts with the sentence “The impaired NO release is also believed to induce diabetes, inflammation which can be prevented by antioxidants.” The sentences are too loose and not clearly linked to one another. Also, regarding the first sentence, it seems reasonable to state that “NO release is believed to be involved in the pathophysiology of diabetes.”
- It would be more appropriate to state that the study “results suggest that compounds in A. bicolor extract could serve as an electron donor, …” than to state that the "extract could serve as an electron donor.”
- It is overly generic to state that “Inflammation often plays a critical role in the pathological progression of organ disease”.

*Response:*

- The first paragraph of the Discussion was rewritten.

*-* By “A. bicolor leaves showed brownish grey and yellow spots”, did the authors mean that the TLC showed “grey and yellow spots”? This statement was clearly written as: " *A. bicolor* leaves showed brownish grey and yellow spots in TLC profiling and which confirming presence of phenol and flavonoids."

- “Cheilanthes albormarginata” was replaced by “Aleuritopteris albomarginata” as per suggestion.

- The paragraph that starts with the sentence “The impaired NO release is also believed to induce diabetes, inflammation which can be prevented by antioxidants.” was reformulate as:

"The impaired nitric oxide (NO) release is believed to contribute to the pathophysiology of diabetes and inflammation, both of which may be mitigated by antioxidants."

- We have revised the statement to clarify that the study "results suggest that compounds in A. bicolor extract could serve as an electron donor," ensuring a more precise interpretation of our findings.

-It is overly generic to state that “Inflammation often plays a critical role in the pathological progression of organ disease” which was rewritten as: Inflammation is a key factor in the progression of various organ diseases, including the heart, pancreas, liver, kidneys, lungs, brain, intestinal tract, and reproductive system, potentially leading to tissue damage and disease.


**Conclusions:**
- It is not necessary to repeat all methods and results covered by the study in the Conclusions. The section should be summarized.
- Which traditional use of A. bicolor does the study support? It is informed in the Introduction that the species is used for gastritis, fever, and wound care, claims that were not assessed in the study.

*Response:*

Thank you for your valuable feedback. We have revised the conclusion as per the reviewer suggestion by omitting the methods, results with correlation with the traditional uses of the A. bicolor as follows:

" In conclusion, this study provides substantial evidence for the diverse therapeutic potential of *Aleuritopteris bicolor* leaf extracts. The extract showed significant antioxidant activity, moderate alpha-amylase inhibition, and notable analgesic and anti-inflammatory effects. These biological activities are likely attributable to the high content of phenolic compounds and flavonoids identified through qualitative and quantitative analyses. These findings not only corroborate the traditional use of *Aleuritopteris bicolor*, but also elucidate its potential as a source of natural compounds for managing pain, inflammation, and potentially diabetes. Further research is warranted to isolate and characterize the active constituents, elucidate their mechanisms of action, and develop novel therapeutic agents derived from this plant. "

We believe these revisions enhance the clarity, scientific rigor, and consistency of the manuscript. Thank you for your thorough feedback.

Thank you.

**Best Regards,**

**Bipindra pandey (Corresponding author)**

**Email:** [bipindra.p101@gmail.com](mailto:bipindra.p101@gmail.com)
