# Supplementary material for: Anti-inflammatory, analgesic, antioxidant, and alpha-amylase inhibitory effects of the hydroethanolic leaf extract of Aleuritopteris bicolor (Roxb.) Fraser-Jenk
Source: PLoS One. 2025 Jun 23;20(6):e0326808. doi: 10.1371/journal.pone.0326808 (PMC12184904; doi:10.1371/journal.pone.0326808)
Supplement: Attachment — Submitted filename: 3rd-Response to Reviewers.docx [file pone.0326808.s005.docx]

**11 March, 2025**

**Respected Academic Editor and Reviewer,**

**Greetings of the day!**

**Thank you for your time and valuable suggestions on our Manuscript (ID:** PONE-D-24-47035R2**) entitled "**Pharmacological Activity of Aleuritopteris bicolor: Anti-inflammatory, Analgesic, Antioxidant, and Alpha-amylase Inhibitory Properties **". We have addressed the reviewer comment and incorporated in the manuscript. We have submitted two separate files of manuscript.**

**1. In the first file, all the revised sentences are highlighted with red color/track changes to show proof of the revision.**

**2. The second file is also the revised file, but the revised sections are not highlighted, and the file is clean here for further process.**

**3. The third file contains the final figure file for the further process.**

**All the revision as requested by the reviewer was addressed and point-to-point answers are given in the response file for each reviewer's questions and comments.**

**I humbly request you to kindly inform me, if you need any further corrections from our side in the future.**

**Thank You.**

**Best Regards,**

**Bipindra pandey (Corresponding author)**

**Email:** [bipindra.p101@gmail.com](mailto:bipindra.p101@gmail.com)

**RESPONSE TO ACADEMIC EDITOR:**

Dear authors, 
I note that you have tried to address the comments raised during the review of this manuscript. However, there are persisting issues that require keen attention. In addition to the reviewer's suggestions, be sure to address the following:

***Editors comments:***

1. The title of your manuscript should be presented concisely. E.g. it can read as 'Anti-inflammatory, Analgesic, Antioxidant, and Alpha-amylase Inhibitory Effects of the hydroethanolic Leaf extract of Aleuritopteris bicolor (Roxb.) Fraser-Jenk.

***Response:***

- Thank you for your insightful feedback regarding the title of our manuscript. We appreciate your suggestion to streamline the title. In response, we have revised the title accordingly "Anti-inflammatory, Analgesic, Antioxidant, and Alpha-amylase Inhibitory Effects of the hydroethanolic Leaf extract of Aleuritopteris bicolor (Roxb.) Fraser-Jenk." to enhance clarity and focus on the study’s strongest findings.

2. The language of presentation should be improved throughout the manuscript- This issue has persisted in your manuscript despite some improvements.

***Response:***

Thank you for your valuable feedback. We acknowledge the need for further improvements in the language and clarity of the manuscript. We have carefully revised the document to enhance readability, ensuring that the language is more precise and professional throughout. We believe these changes address the concerns raised and improve the overall presentation.

3. The statement ' Powdered A. bicolor leaves were soaked in an 80% (v/v) ethanol solution at a ratio of 1:8 for 3 days in an amber colored 
glass container.' under preparation of A. bicolor leaves extract is ambiguous. If the powder was soaked in an 80% (v/v) ethanol:water solution, then what does the ratio 1:8 mean? Clarify this confusion! Also, briefly explain why this solvent system was was chosen for extraction. Also, considering the high boiling point of ethanol and water used to make the extraction solvent, is a rotary evaporation temperature of 40 oC sufficient to remove the solvents? You should also include an appropriate reference for the extraction procedure.

***Response:***

Thank you for your helpful feedback. We have clarified the statement by specifying that the ratio of 1:8 refers to the mass of powdered A. bicolor leaves to the volume of the 80% ethanol solution. We have also added an explanation for choosing this solvent system, highlighting its efficacy in extracting both polar and non-polar compounds from plant material. Regarding the rotary evaporation, we have clarified that 40°C is a suitable temperature to remove the solvents while maintaining the integrity of the extract. Additionally, we have included a reference to support the extraction procedure.

4. Include appropriate references for all the experimental methods, and briefly describe the procedures, to facilitate reproducibility.

**Response:** Thank you for your valuable suggestion. We have now included appropriate references for all experimental methods and provided brief descriptions of the procedures to enhance clarity and ensure reproducibility.

5. In TLC profiling, the rationale for using chloroform:methanol:water; 7:3:0.5 ratio is unclear- Why this ratio and not any other?

***Response:***

Thank you for your comment. The chloroform:methanol:water (7:3:0.5) ratio was selected based on preliminary trials to achieve optimal separation of the phytoconstituents present in Aleuritopteris bicolor. During the pilot testing, this ratio provided the best resolution and distinct band separation compared to other tested solvent systems and which included the less polar to more polar type compound separation. We have included this rationale in the manuscript for clarity.

6. Tables 2 and 3: Did you perform any inferential statistics for these results? This is crucial for drawing objective conclusions from these findings, and therefore must be addressed.

***Response:***

Thank you for your insightful comment. We acknowledge the importance of inferential statistics for drawing objective conclusions for the Table 2 and 3. However, due to the varying radical scavenging properties of different antioxidant methods (DPPH, NO, H_2_O_2_, FRAP) only a single method was compared using descriptive statistics by calculating the mean and standard error of mean for data reliability and comparison. This approach was chosen to highlight the trend in antioxidant activity rather than direct statistical inference across different assays. Likewise, for the Table 3, the alpha-amylase activity of A. bicolor was compared with acarbose in different concentration was compared by the calculating descriptive statistics. We have clarified this in the manuscript accordingly. If required any further inferential statistics please guide us in applying such test.

7.Tables 4 and 5 results: It is unclear how the comparison was performed, row-wise or column-wise? There is an effect of dose at each timepoint, and the effect of each treatment across time!

***Response:***

Thank you for your valuable comment. We have clarified in the manuscript that comparisons in Table 4 and 5 were performed both row-wise (to assess the effect of each treatment over time) and column-wise (to evaluate the effect of different doses at each time point). Additionally, we have discussed the dose-dependent effect at each time point and the variation in analgesic activity across different time intervals in each result section.

8. The discussion should be improved by highlighting the rationale of each experiment, comparing your findings with those of other scholars, and explaining the reasons for similarities/differences thereof, and implications.

***Response:***

Thank you for your insightful comments. We have revised the discussion section to better highlight the rationale behind each experiment. Additionally, we have made comparisons with relevant studies and provided explanations for any similarities or differences observed in our findings. The implications of these comparisons have also been clearly addressed to strengthen the discussion.

**RESPONSE TO REVIEWER:**

Reviewers' comments:

Reviewer's Responses to Questions

**Comments to the Author**

1. If the authors have adequately addressed your comments raised in a previous round of review and you feel that this manuscript is now acceptable for publication, you may indicate that here to bypass the “Comments to the Author” section, enter your conflict of interest statement in the “Confidential to Editor” section, and submit your "Accept" recommendation.

Reviewer #2: All comments have been addressed

Reviewer #3: All comments have been addressed

2. Is the manuscript technically sound, and do the data support the conclusions?

The manuscript must describe a technically sound piece of scientific research with data that supports the conclusions. Experiments must have been conducted rigorously, with appropriate controls, replication, and sample sizes. The conclusions must be drawn appropriately based on the data presented.

Reviewer #2: Yes

Reviewer #3: Yes

3. Has the statistical analysis been performed appropriately and rigorously?

Reviewer #2: Yes

Reviewer #3: Yes

4. Have the authors made all data underlying the findings in their manuscript fully available?

The [PLOS Data policy](http://track.editorialmanager.com/CL0/http:%2F%2Fwww.plosone.org%2Fstatic%2Fpolicies.action%23sharing/1/010f01956f72e526-8ee8134c-289d-4077-bac0-e729f774c9dd-000000/iXYDKfTcrI6anz1GUzMPOwJ-aSMv7MvS76q0mAdfn28=201) requires authors to make all data underlying the findings described in their manuscript fully available without restriction, with rare exception (please refer to the Data Availability Statement in the manuscript PDF file). The data should be provided as part of the manuscript or its supporting information, or deposited to a public repository. For example, in addition to summary statistics, the data points behind means, medians and variance measures should be available. If there are restrictions on publicly sharing data—e.g. participant privacy or use of data from a third party—those must be specified.

Reviewer #2: Yes

Reviewer #3: Yes

5. Is the manuscript presented in an intelligible fashion and written in standard English?

PLOS ONE does not copyedit accepted manuscripts, so the language in submitted articles must be clear, correct, and unambiguous. Any typographical or grammatical errors should be corrected at revision, so please note any specific errors here.

Reviewer #2: Yes

Reviewer #3: Yes

6. Review Comments to the Author

Please use the space provided to explain your answers to the questions above. You may also include additional comments for the author, including concerns about dual publication, research ethics, or publication ethics. (Please upload your review as an attachment if it exceeds 20,000 characters)

**Reviewer #2:**

In my opinion, the manuscript “Pharmacological Activity of Aleuritopteris bicolor: Anti-inflammatory, Analgesic, Antioxidant, and Alpha-amylase Inhibitory Properties” is well written and presents interesting information regarding the use of Aleuritopteris bicolor leaves and can be accepted for publication. If possible, information regarding the consumption of this leaf and the related issue could be added.

***Response to Reviewer 2:***

We sincerely appreciate the reviewer’s positive feedback on our manuscript. We acknowledge that there is limited research on Aleuritopteris bicolor leaves. However, we have included some related medicinal uses of Aleuritopteris bicolor fronds in the Introduction section to provide context. Thank you for your valuable suggestion.

**Reviewer #3:**

I consider the authors have responded to all review comments accordingly. As suggested by the reviewer, the authors have improved the manuscript writing. Therefore, I would recommend the article for publication in Plos One, but I am attaching a few language suggestions/corrections that need to be addressed first:

***Response to Reviewer 3:***

**Introduction:**

Page 4: In the sentence “Non-steroidal anti-inflammatory drugs… manage pain”, substitute “is” with “are.”

***Response:*** The verb "is" has been replaced with "are" in the sentence "Non-steroidal anti-inflammatory drugs… manage pain" as suggested.

Page 4: In the sentence “In spite of this, some literature reports…”, delete the word “some.”

***Response:*** The word "some" has been deleted from the sentence "In spite of this, some literature reports…" for clarity.

Page 5: Refine the sentence “Since for searching the safe and effective alternative drug candidates from the natural products is necessary for treating the inflammation and pain.”

***Response:*** The sentence has been refined for clarity and conciseness. The revised version ensures better readability and precision.

Page 5: Substitute the word “modifaction” with “modification.”

***Response:*** The typo "modifaction" has been corrected to "modification."

**Discussion:**

Page 23: Substitute the excerpt “an electron donor” with “electron donors.”

***Response:*** The excerpt “an electron donor” has been replaced with “electron donors” as suggested.

We believe these revisions enhance the clarity, scientific rigor, and consistency of the manuscript. Thank you for your thorough feedback.

Thank you.

**Best Regards,**

**Bipindra pandey (Corresponding author)**

**Email:** [bipindra.p101@gmail.com](mailto:bipindra.p101@gmail.com)
