# Supplementary material for: Anti-inflammatory, analgesic, antioxidant, and alpha-amylase inhibitory effects of the hydroethanolic leaf extract of Aleuritopteris bicolor (Roxb.) Fraser-Jenk
Source: PLoS One. 2025 Jun 23;20(6):e0326808. doi: 10.1371/journal.pone.0326808 (PMC12184904; doi:10.1371/journal.pone.0326808)
Supplement: Attachment — Submitted filename: 4th-Response to Reviewers.docx [file pone.0326808.s006.docx]

**22 March, 2025**

**Respected Academic Editor,**

**Greetings of the day!**

**Thank you for your time and valuable suggestions on our Manuscript (ID:** PONE-D-24-47035R3**) entitled "**Anti-inflammatory, Analgesic, Antioxidant, and Alpha-amylase Inhibitory Effects of the Hydroethanolic Leaf Extract of *Aleuritopteris bicolor* (Roxb.) Fraser-Jenk**". We have try to addressed all the concerns raised during in the previous reviewing process and incorporated in the manuscript. We have submitted two separate files of manuscript.**

**1. In the first file, all the revised sentences are highlighted with red color/track changes to show proof of the revision.**

**2. The second file is also the revised file, but the revised sections are not highlighted, and the file is clean here for further process.**

**3. The third file contains the final figure file for the further process.**

**All the revision as requested by the reviewer was addressed and point-to-point answers are given in the response file for each reviewer's questions and comments.**

**I humbly request you to kindly inform me, if you need any further corrections from our side in the future.**

**Thank You.**

**Best Regards,**

**Bipindra pandey (Corresponding author)**

**Email:** [bipindra.p101@gmail.com](mailto:bipindra.p101@gmail.com)

**RESPONSE TO ACADEMIC EDITOR:**

Dear Authors,

I have assessed your article and note that you made some of the requested modifications. However, there are some outstanding issues, which must be resolved before can acceptable for publication. The results should be analyzed statistically, as I suggested previously. Please note that without appropriate statistics, we cannot interpret correctly and the conclusions regarding significance cannot be substantiated. Besides, revise the methods and ensure they are technically correct and scientifically justified. For instance, there is a conflict between the extraction and TLC procedures and conventional protocols. The choice of the mobile phase in your TLC may not be appropriate, and this can be evinced by the photographs you attached. Revise the grammatical errors throughout the manuscript and ensure your ideas flow logically.

***Comments 1:*** TLC profiling

In TLC profiling, the rationale for using chloroform:methanol:water; 7:3:0.5 ratio is unclear- Why this ratio and not any other? For instance, there is a conflict between the extraction and TLC procedures and conventional protocols. The choice of the mobile phase in your TLC may not be appropriate, and this can be evinced by the photographs you attached.

***Response:***

Thank you for your comment. We have revised the methodology to ensure technical accuracy and consistency with conventional protocols, particularly in the extraction, TLC procedure. TLC solvents choses based on the hit and trial methods with various solvents ratio and the best optimized solvent was chloroform:methanol:water (6:4:1) which was also written as in the TLC chromatogram for remembering during the experiments procedure. Respected editor, I have gone through all the raw data and checked it once and confirmed. There is some typo graphical errors in the previous manuscript file. We have included revised solvent system ratio and choosing this rationale in the manuscript for clarity.

***Comments:*** Statistical test

In Tables 2 and 3 Did you perform any inferential statistics for these results? This is crucial for drawing objective conclusions from these findings, and therefore must be addressed.

***Response:***

Thank you for your insightful comment. We acknowledge the importance of inferential statistics for drawing objective conclusions for the Table 2 and 3 and performed statistical analysis on the results to ensure proper interpretation and validation of significance. The antioxidant and alpha-amylase inhibitory activities mean value of the *A. bicolor* hydroethanolic extract with standard drug were evaluated by using the Independent T-test. Likewise descriptive statistics of different antioxidant methods (DPPH, NO, H_2_O_2_, FRAP) and alpha amylase inhibitory activity is also evaluated by calculating the IC50 value and mean, standard error of mean for data reliability and comparison. Further details were changes in the revised manuscript accordingly in both methodology and results sections as per your suggestion.

***Comments:***

Revise the grammatical errors throughout the manuscript and ensure your ideas flow logically.

***Response:***

Thank you for your valuable feedback. We have try to carefully revised grammatical errors and also try to enhance the clarity and logical flow of the manuscript.

We believe these revisions enhance the clarity, scientific rigor, and consistency of the manuscript. Thank you for your thorough feedback.

Thank you.

**Best Regards,**

**Bipindra pandey (Corresponding author)**

**Email:** [bipindra.p101@gmail.com](mailto:bipindra.p101@gmail.com)
